# Is 10 minutes sufficient? Follow-up consultation in Ecuadorian Social Security health systems

**DOI:** 10.3389/fpubh.2026.1816758

**Published:** 2026-04-10

**Authors:** Doris Quiroz-Cárdenas, Fiorella Quiroz-Cárdenas, José Francisco López-Gil

**Affiliations:** 1 Centro de Salud Chimbacalle, Ministerio de Salud Pública, Quito, Ecuador; 2 School of Medicine, Universidad Espíritu Santo, Samborondón, Ecuador; 3 Vicerrectoría de Investigación y Postgrado, Universidad de Los Lagos, Osorno, Chile

**Keywords:** consultation length, quality of care, patient safety, medical systems, Ecuador

On February 20, 2026, the Ecuadorian Social Security Institute introduced a system change, reducing appointment duration to as little as 10 min for specific visit types (follow-up test review, prescription renewal), while maintaining 20-min slots for non-surgical specialty visits, as an efficiency measure to increase the number of patients seen per day. Although Ecuadorian Social Security Institute officials have framed this as a reorganization rather than a reduction, arguing that appointment length ultimately depends on the physician's clinical judgment, the policy establishes a structural default that warrants scrutiny (1, 2). This policy deserves scrutiny not as a narrow administrative change, but as an issue related to the safety, continuity, and effectiveness of patient care (3). Consultation length is a structural determinant of care quality. Evidence from primary care and outpatient settings consistently links shorter visits to lower patient satisfaction, reduced opportunity for shared decision-making, and higher rates of potentially preventable hospitalizations (4). Compressing complex clinical encounters into 10 min risks undermining diagnostic accuracy, monitoring of therapeutic response, review of complementary studies, and prevention of complications. Patients with multiple chronic diseases require comprehensive assessment, prioritization of problems, dynamic therapeutic adjustment, and shared decision-making, processes that require adequate clinical time (5), which is associated with better outcomes and lower long-term costs (6, 7).

The burden of these constraints falls disproportionately on clinicians and, through them, on patients. Task saturation and time pressure are recognized drivers of physician burnout (8, 9). When consultation time is reduced without a commensurate reduction in clinical complexity or administrative load, burnout and attrition are likely to be associated with medical errors and adverse events (10, 11). As well burnout can exacerbate workforce shortages, creating a vicious cycle: fewer clinicians, more pressure on those who remain, and further erosion of the conditions needed for safe, effective care.

Ecuador's case sits within a broader Latin American pattern. Public health systems across the region face chronic underfunding, workforce shortages, demand that outstrips capacity (12, 13), and prolonged waiting periods to access to specialized health services (14). For instance, Colombia, despite having 2.5 physicians per 1,000 inhabitants and lower-than-average satisfaction with healthcare quality (15), mandates 20-min appointments per patient (16). Similarly, Chile has 3.3 physicians per 1,000 inhabitants, low satisfaction rates, and 15-min appointment schedules (17). In this context, policies that prioritize productivity, more consultations per clinician per day, over adequacy of contact time are a regionally recurrent pattern, one that Ecuador now reproduces. Such approaches may improve headline ‘'access” metrics while obscuring declines in the quality and safety of care (18). The right to health encompasses both access and quality. When access is compromised to inflate the former, the right is undermined (9, 19).

We do not dismiss the real pressures on policymakers. Waiting lists and population expectations create legitimate pressure to see more patients. Nevertheless, solving capacity problems by compressing time per patient is a high-risk strategy. It undermines the bioethical principles of primary health care, particularly the principle of non-maleficence (20), which involves avoiding preventable harm through preventive interventions, the promotion of healthy lifestyles, and health education.

## Recommendations

First, consider the length of subsequent consultations as an indicator of quality in patient care. Minimum consultation length standards should be evidence-based and depend on morbidity and mortality, not just on productivity targets.

Second, consider longer consultation time as a way to avoid errors in diagnosis and therapeutic follow-ups that could lead to medical-legal risks. Also, adequate time allows for active communication with patients.

Third, invest in staff to reduce the administrative burden on physicians so that the time available can be used for clinical work that promotes a meaningful physician-patient relationship.

Fourth, situate national decisions in regional and global experience. Latin American and international evidence on consultation length, burnout, and quality should inform policy so that lessons from one country inform others.

The measure adopted by Ecuador is an example of how social security institutions respond to productivity pressures by shortening consultation times and sacrificing quality of care and patient satisfaction. This tension is also an ethical one. Adequate consultation time is a precondition for informed consent and shared decision-making, cornerstones of patient autonomy. When time constraints prevent clinicians from explaining diagnoses, discussing treatment options, or addressing patient concerns, the therapeutic relationship is reduced to a transactional encounter. Clinicians, in turn, face the professional and moral burden of being asked to deliver care they know to be inadequate, a conflict that erodes professional integrity and contributes to burnout. Policymakers therefore bear ethical responsibility not only for access metrics but for the conditions under which care is actually delivered. The aim must be access with quality. Otherwise, the right to health remains unmet.
